# Impact of Thyme Microcapsules on Histamine Production by *Proteus bacillus* in Xinjiang Smoked Horsemeat Sausage

**DOI:** 10.3390/foods10102491

**Published:** 2021-10-18

**Authors:** Honghong Yu, Yali Huang, Liliang Lu, Yuhan Liu, Zonggui Tang, Shiling Lu

**Affiliations:** 1 Laboratory of Meat Processing and Quality Control, College of Food Science and Technology, Shihezi University, Shihezi 832000, China; Yhh195812@163.com (H.Y.); hyl2026@163.com (Y.H.); 2 Analysis and Testing Center, Xinjiang Academy of Agriculture and Reclamation Science, Shihezi 832000, China; lubeyond@126.com (L.L.); yuhanliu0815@163.com (Y.L.); zongguitang@163.com (Z.T.)

**Keywords:** thyme microcapsules, *Proteus bacillus*, histamine, histidine decarboxylation pathway, smoked horsemeat sausage

## Abstract

Here, we explored the influences of thyme microcapsules on the growth, gene expression, and histamine accumulation by *Proteus bacillus* isolated from smoked horsemeat sausage. RT-qPCR was employed to evaluate the gene expression level of histidine decarboxylase (HDC) cascade-associated genes. We used HPLC to monitor histamine concentration both in pure culture as well as in the processing of smoked horsemeat sausage. Results showed that histamine accumulation was suppressed by thyme microcapsule inhibitory effect on the histamine-producing bacteria and the reduction in the transcription of hdcA and hdcP genes. Besides, compared with thyme essential oil (EO), thyme microcapsules exhibited higher antibacterial activity and had a higher score for overall acceptance. Therefore, the addition of thyme microcapsules in Xinjiang smoked horsemeat sausage inhibits histamine accumulation.

## 1. Introduction

Histamine is a low molecular weight nitrogenous organic compound that plays a pivotal role in human learning as well as memory, body temperature, and immune responses [1]. Histamine oxidases quickly degrade the exogenous histamine that is ingested in food under normal conditions; however, when the process of detoxification is insufficient, or the concentrations of histamines present in food are very high, the histamine might lead to histamine intolerance or intoxication [2,3]. A food-poisoning incidence reported in January 2009 that was associated with histamine toxicity caused illness in 53 persons [4]. The Xinjiang smoked horsemeat sausage made via spontaneous fermentation is popular for its appetizing sensory features as well as excellent nutritional properties [5]. Nevertheless, the traditional sausage is often produced in small-scale plants after a process of spontaneous fermentation [6], which more easily leads to the accumulation of high levels of biogenic amines (BAs), e.g., putrescine, tyramine, as well as histamine [7]. In fermented meat products, histamine is formed through histidine decarboxylation mediated by the histidine decarboxylase enzymes originating from the bacteria present in food [8]. The bacterial histidine decarboxylases have been broadly studied and characterized in various organisms [9], and two distinct enzyme classes have been differentiated, namely pyridoxal phosphate dependent as well as the pyruvoyl dependent. The diverse species of gram-negative bacteria, especially the lactic acid bacterial species involved in food spoilage or fermentation possess, the pyridoxal phosphate-dependent decarboxylases, whereas the gram-positive bacteria possess the pyruvoyl-dependent decarboxylases [10]. Studies have shown that the histidine genes clusters related to histidine decarboxylase (HDC) consist of 4 ORFs, hdcA, hdcRS, hdcP, as well as hdcB, and their sequences are highly conserved in numerous lactic acid bacteria. The hdcA OFR codes for the histidine decarboxylase, hdcP encodes a histidine/histamine protein, whereas hdcRS encodes a histidyl-tRNA synthetase; nonetheless, the role of the hdcB gene is still unclear [8,11].

The histamine content in fermented sausages depends on many factors, including the existence of microorganisms, which decarboxylate the amino acids, as well as a conducive environment for the growth of decarboxylase-producing microbes [12]. Furthermore, due to its heat stability, once histamine is formed, it is difficult to destroy with high-temperature treatment [13]. Hence, microbial food contamination should be prevented to minimize the possibility of histamine production [5,14]. Therefore, varieties of antibacterial agents have been applied in food to prevent the histamine aggregation. Thyme oil obtained from *Thymus vulgaris* L. has been opined to possess elevated antimicrobial, insecticidal, as well as phytotoxic features owing to the phenolic constituents, particularly carvacrol (2-methyl-5-methyl ethyl)-phenol) as well as thymol (5-methyl-2 (1methylethyl) phenol) [15,16]. However, the application of thyme essential oil (EO) in food preservation is limited because it is likely to change the original flavor of food products. Moreover, when the oil is exposed to heat, oxygen, or light, it is highly susceptible to oxidative deterioration [17]. The technology of encapsulation provides an efficient approach to stabilize EOs as well as impede volatile ingredients loss [18]. Encapsulation of cinnamon EO is achieved through the inclusion complexation approach using the β-cyclodextrin and has high antifungal bioactivity against *Botrytis* spp. [17]. Ginger EO microcapsules, with a particle size of 8.2–15.3 μm, were encapsulated by gum arabic (GA) and/or maltodextrin (MD) through spray drying and possess effective antioxidant activity, extending the shelf life of the food products [19]. Hence, EO microcapsules confer antibacterial and antioxidants effects, with prospective application in meat products

However, to the best of our knowledge, there is a knowledge gap in the application of thyme microcapsules in sausages. Hence, the purpose of the research was to assess if microcapsules of thyme affect the accumulation of histamine, the growth of microorganisms, transcription of histidine decarboxylase (HDC) cascade-associated genes, and the sensory quality of smoked horsemeat sausage.

## 2. Materials and Methods

### 2.1. Microorganisms and Growth Conditions

A previously isolated histamine-producing microorganism, *Proteus bacillus,* from naturally smoked horsemeat sausage in our laboratory, was used in this study (Animal Product Processing Laboratory, Shihezi, China). The sequences were deposited in GenBank databases under accession number MN483275. *Proteus bacillus* was grown on Brain-Heart Infusion Broth (BHI) media, enriched with 100 Mm histidine (Sigma, Santa Clara, CA, USA) at 37 °C. The strain used in this work had been previously identified by molecular methods [20]. Overnight cultures of *P. bacillus* strains were used as inoculum for all fermentation assays. Each fermentation experiment was performed using the same stock culture medium and overnight culture inoculants to ensure the same CFU/mL (10^5^–10^6^). Thyme microcapsules and thyme essential oil were supplied by the Boao Extension Technology Co. Ltd. in Beijing. Five batches were prepared (10 mL): a batch without histidine (control); histidine and 0% thyme microcapsules (0% microcapsules); histidine and minimum inhibitory concentration (MIC) thyme microcapsules (MIC microcapsules); histidine and 1/2 MIC thyme microcapsules (1/2MIC microcapsules); and histidine and the same amount of essential oil as microcapsules (essential oil). Brain-Heart Infusion Broth (BHI) media (10 mL) required the following ingredients: beef brain (20%), beef heart infusion juice (25%), peptone (1%), glucose (0.2%), NaCl (0.5%), and agar (2%). Brain-Heart Infusion Broth (BHI) media was obtained from AoBoXing Company (Beijing, China). Samples were collected (2 mL) every four hours for 48 h. To check the microbial growth in all cultures, we measured the absorbance at 600 nm (OD600) using a multi-modal reader (Bio Tek, Unalaska, AK, USA). 

### 2.2. Determination of Relevant Indicators in a Pure Culture System

The pH of the samples was measured using a Sartorius UB pH-meter (Sartorius, Gogenting, Germany). We calibrated the pH meter with phosphate buffer and potassium hydrogen phthalate buffer. After the calibration was completed, we washed the composite electrode with distilled water and dried the filter paper and inserted it into the sample solution to ensure that the glass bulb at the front of the electrode was in uniform contact with the standard buffer solution. After the pH meter stabilized, showing the pH value of the unknown solution, we pressed the “OK” button to measure the solution again [2]. The measurement accuracy of the pH meter is 0.01. *P bacillus* MN483275 was cultured in BHI medium and supplemented with 100 Mm histidine to activate the transcription of the hdc gene [21]. To harvest the cells, we centrifuged the broth culture after 24 h of growth at 10,000× *g* for 3 min at 4 °C and resuspended in 1 mL of TRIzol reagent. TRIzol reagent (Sigma, Santa Clara, CA, USA) was used to extract total RNA, as previously described by del Rio et al. [22]. Sample supernatants were obtained by centrifugation at 5000× *g* for 10 min at 4 °C. High-performance liquid chromatography (HPLC) was used to determine histamine concentration [5]. Three biological replicates were performed for each experiment.

### 2.3. Sausage Sample Preparation

The horse meat was cut into cubes about 1 cm^3^ in size. Four batches of smoked horsemeat sausage were processed: a control batch without *P. bacillus* or thyme microcapsules (batch CK); a batch inoculated with *P. bacillus* (batch P); a batch inoculated with *P. bacillus* and thyme microcapsules (batch PMT); and a batch inoculated with *P. bacillus* and thyme essential oil (batch PO). Sausage preparation (10 kg/batch) required the following ingredients: basic raw materials of lean horsemeat (80%) and fat horsemeat (20%); and auxiliary raw materials of pepper (0.1%), white sugar (2%), sodium chloride (2.5%), ginger powder (0.2%), monosodium glutamate (0.1%), spiced powder (0.1%), star anise (0.1%), and a smoke solution (1%). Thyme microcapsules and thyme oil were added at a concentration of 0.156%. Each batch was kept at 4 °C for 24 h and then stuffed (300–350 g/sausage) into a natural casing (horse’s small intestine) previously soaked in salt water and with a diameter of 5–6 cm [3]. The culture of *P. bacillus* was suspended in sterile water (100 mL) to attain a final level of 10^4^ CFU/g of sausage. Fermentation and ripening of the sausages was performed at a steady humidity as well as temperature in an incubator (DNP-9272, Jinghong Company, Shanghai, China) at 90–95% relative humidity (RH) and 18–20 °C first for 2 days and then at 75–80% RH for 26 days at 10–12 °C [23]. We collected the samples at 0 (initial sausages), 3 (after fermentation), 7, 14, 21, as well as 28 days.

### 2.4. Microbial Counts

The bacterial numbers were enumerated during the 28 days fermentation period using a previously document protocol [5] with slight adjustments. Using aseptic techniques, 10 g of each sample was homogenized in 90 mL of sterile saline (containing 0.85% NaCl), mixed in a stomacher for 10 min at 250 rpm, and the suspension was diluted serially (1:10) using sterilized saline in triplicates [24]. This was followed by inoculation of the diluted suspensions in different agars: gram-positive catalase-positive cocci (GCC+) were examined using mannitol salt agar (MSA) medium at 37 °C for 48 h; *Enterobacteriaceae* were analyzed using Violet Red Bile Glucose Agar (VRBGA) for 48 h at 37 °C, whereas lactic acid bacteria (LAB) were determined on de Man Rogosa Sharpe agar (MRS) medium at 30 °C for 2 days [5]. AoBoXing Company, Beijing, China provided all the growth media.

### 2.5. RNA Extraction

The RNA from experimental sausages was isolated, as per protocol documented by del Rio et al. [22] with slight adjustments. In brief, 1 mL of Trizol was added to 100 mg of shredded sausages in a 1.5-mL centrifuge tube, followed by homogenization, and left to stand for 5 min at room temperature. Chloroform (0.2 mL) was introduced into the homogenate and then mixed through, shaking for 15 s, then left standing for another 2 min. Next, we spun the mixture for 10 min at 12,000× *g* at 4 °C and then aliquoted the supernatant into clean tubes. We added 0.5 mL isopropanol into the mixtures, gently mixed, and maintained standing for 10 min at room temperature. After that, 10-min centrifugation of the mixture was performed at 12,000× *g* at 4 °C, and the supernatant was discarded. The resulting precipitate was rinsed using 1 Ml 75% ethanol to precipitate it further. We air-dried the precipitate and re-suspended in DEPC-treated H_2_O. The RNA was inoculated with DNase (ABM, Vancouver, BC, Canada) to degrade any contaminating DNA. The concentration of the RNA was estimated by measuring its absorbance at 260 nm as well as 280 nm on a NanoDrop ND-2000c UV/vis Spectrophotometer (Thermo Scientific, Shanghai, China).

### 2.6. Assessment of RNA Levels by RT-qPCR

The 5X All-In-One RT Master Mix (AccuRT Genomic DNA Removal Kit) (ABM, Vancouver, BC, Canada) was employed to convert the RNA into cDNA via reverse transcription. Thereafter, qPCR was performed using the Stratagene MX 3000 sequence detection system (Agilent Technologies, Santa Clara, CA, USA) in 25 μL reaction mixture, as documented previously [25]. The reaction mixture contained the primers as well as the EvaGreen 2X qPCR Master Mix, which uses low ROX as a passive reference (ABM, Vancouver, BC, Canada). The specific primers utilized are listed in Table 1. The hdcA-F/hdcA-R as well as hdcP-F/hdcP-R primer pairs targeted the hdcA (the first gene of the HDC cluster) and hdcP (encoding for histidine/histamine exchanger) [11]. The hdcRS-F/hdcRS-R as well as hdcB-F/hdcB-R primer pairs [9] targeted the hdcRS gene (encoding the histidyl-tRNA synthetase) and hdcB (whose role is unclear), respectively [8]. The tufF/tufR and recA-F/recA-R primer pairs [9] targeted the thermo-unstable elongation factor (tuf) as well as the RNA polymerase alpha-subunit (recA) genes, respectively, which served as the reference genes. Negative controls were included as samples without DNA in each run. The 2^−ΔΔCt^ approach was employed to determine the relative gene-expression level [26]. RT-qPCR was conducted on RNA samples extracted from 3 different cultures for each condition.

### 2.7. Histamine Determination from Sausage Samples

The aggregated histamine in the experimental sausages was isolated using a previously described protocol [5] with slight adjustments. We homogenized 5 g of the sausages using an ULTRA-TURRAX T25 basic ZKA (WERKE, Sasel, Hamburg, German) in 20 mL of 0.4 M perchloric acid. The mixture was centrifuged for 10 min at 5000× *g* at 4 °C and collected the supernatant. (AllegraX-22, Santa Clara, CA, USA). The volume of the filtrate was adjusted to 50 mL using 0.4 M perchloric acid. Derivatization of biogenic amines was conducted using dansyl chloride according to the protocol documented by Lu et al. and Sun et al. [5,27]. We put the sample extract (1 mL) into a 5-mL volumetric flask and then added sodium hydroxide (2 N, 200 mL), saturated sodium bicarbonate (300 mL), and dansyl chloride (10 mg/mL; Sigma, CA, USA) to the volumetric flask and incubated at 40 °C in the dark for 45 min. To remove residual dansyl chloride, we added 100 mL of ammonia, incubated at room temperature for 30 min, adjusted the volume of the reaction mixture to 5 mL with acetonitrile, and centrifuged at 3000× *g* for 5 min [5]. For HPLC analysis, a 0.45-μm membrane syringe filter was employed to filter the supernatant. Histamine content was detected by HPLC (LC-2010AHT, Shimadzu Corporation, Beijing, China) by a C18 column (Spherisob, 2.5 μm octadecylsilane, 250-mm 94.6-mm internal diameter), an injection volume of 10 μL, a flow rate of 0.8 mL/min, as well as column temperature of 35 °C. The mobile phase was composed of ultrapure water (eluent A) and acetonitrile (eluent B), and the gradient program was 40% A + 60% B at 0 min; 30% A + 70% B at 5 min; 10% A + 90% B at the 10th minute; 100% B at the 15th minute; and 40% A + 60% B at the 25th minute. The 1.00 mg/L histamine standard solution was appropriately diluted with acetonitrile and then derivatized and determined according to the method of Park et al. [28]. The detection limit (RSN = 3) and the limit of quantification (RSN = 10) were determined by the signal-to-noise ratio. Histamine was assayed at 254 nm. Moreover, all analytical determinations were performed in triplicate for each sausage sample.

### 2.8. Sensory Evaluation

The sensory quality of the smoked horsemeat sausages was assessed at the end of the fermentation, as previously documented, with slight modifications [27]. The sensory analysis was carried out using a sensory panel of 10 investigators, comprising of 5 females as well as 5 males. The assessors were selected based on their sensory potential and prior experience in performing a sensory assessment of meat products (Table 2). Each panel investigator was tasked with rating the appearance (color, gloss, dry), flavor (sourness, odor), and texture (hardness, organizational structures) of the sausage samples. Attributes were quantified with a numerical intensity scale from 0 to 7, where 0 = attribute not detected, and 7 = attribute very intense [29]. The total scores for each sample were calculated by adding the average score given by each panel member for each of the seven attributes.

### 2.9. Statistical Analyses

The Origin 8.5 software (Origin Lab) was utilized for data analysis. Means ± standard deviations were computed from three independent replicates. The SPSS software 23 package (IBM, Armonk, NY, USA) was employed to evaluate the differences between groups. *p* < 0.05 signified statistical significance.

## 3. Results and Discussion

### 3.1. The Thyme Microcapsules Effect on P. bacillus Growth and Histamine Generation

**Figure 1:**
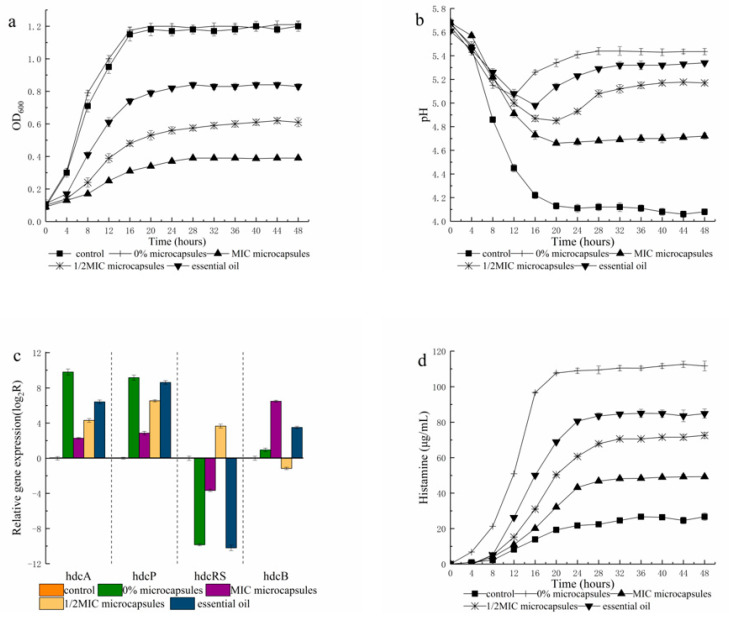
Effect of thyme microcapsules on *P. bacillus* growth (**a**), pH (**b**), gene expression of HDC cluster (**c**) and histamine accumulation (**d**) during 48 h. Batch Control: without histidine (control); 0% microcapsules: histidine + 0% microcapsules; MIC microcapsules: histidine + MIC microcapsules; 1/2MIC microcapsules: histidine +1/2MIC microcapsules; essential oil: histidine + essential oil.

As indicated in (Figure 1a), the absorbance values (at 600 nm) of the microbial cultures treated with thyme oil was consistently lower than that of control batch at 48 h, suggesting that thyme essential oil (EO) repressed *P. bacillus* growth. Moreover, compared with the thyme oil group, thyme microcapsules exhibited higher antibacterial bioactivity [18,30]. However, thyme microcapsules only prolonged the life of the strain but did not change the growth pattern. Thyme microcapsule at varied levels had different suppressive influences on the strain (*p* < 0.05), and microbial growth decreased with escalating thyme microcapsules.

The changes in pH over 48 h in all batches are shown in Figure 1b. The pH values decreased in the first 12 h and stabilized gradually after 20 h in the control batch, indicating that the microbes started to grow rapidly, and the products of fermentation were accumulating slowly. In the histidine-treatment batches, the microbial culture pH values (0% microcapsules, MIC microcapsules, 1/2 MIC microcapsules and essential oil) were higher relative to control batch, attributable to the aggregation of alkaline substances, such as histamine.

The thyme microcapsules group’s pH was lower compared to the other groups, which revealed that thyme microcapsules inhibit *P. bacillus* growth as well as the HDC gene cluster expressions, thereby reducing the histamine accumulation.

The histamine standard solution with a mass concentration of 1.00 mg/L was diluted into different low-mass concentration solutions, and samples were injected for determination after derivatization. The results showed that when the RSN was 3, the mass concentration of histamine standard solution was 0.03 mg/L; when the RSN was 10, the mass concentration of histamine standard solution was 0.12 mg/L. Therefore, the detection limit of the instrument was 0.03 mg/L, and the limit of quantification was 0.12 mg/L. The accumulation of histamine over 48 h in all batches is shown in Figure 1d. Histamine was detected at 4 h and was found to gradually stabilized after 28 h in the control batch. In the histidine treatments, the histamine levels in the thyme microcapsules batches (MIC microcapsules and 1/2 MIC microcapsules) were significantly lower (*p* < 0.05) in contrast with the other batches (control and 0% microcapsules). Additionally, the thyme microcapsules influence on histamine accumulation was higher compared to that of thyme EO, suggesting that thyme microcapsule significantly decreased the accumulation of histamine. Moreover, the biosynthesis of histamine decreased with increased concentration of thyme microcapsules. The accumulation of histamine may confer resistance to acid stress acquired via the consumption of intracellular protons through a decarboxylation process, as has been documented in other bacterial species [31,32,33]. Thyme microcapsules suppressed histamine accumulation by inhibiting the histamine-producing bacteria and the expression of hdcA as well as hdcP genes.

### 3.2. Gene Expression in Pure Culture

The transcription of the HDC gene cluster is shown in Figure 1c. The HDC gene cluster includes the hdcA gene, histidyl-tRNA synthetase (hdcRS), a transporter (hdcP), as well as the hdcB gene, with the four genes oriented in a similar direction [34]. The comparative assessment showed that the expression of hdcB as well as hdcRS gene was not modulated by thyme microcapsule levels in the growth medium. However, the hdcA and hdcP gene expressions were remarkably repressed by increased thyme microcapsule levels. The highest expression activity of the hdcA gene was reported at 0% thyme microcapsules (181.02 times the expression in MIC thyme microcapsules), and the highest transcriptional activity of the hdcP gene was reported at 0% thyme microcapsules (78.25 times the expression in MIC thyme microcapsules). The hdcA gene encodes a 377 amino acid polypeptide and is believed to be a pyridoxal-P-dependent histidine decarboxylase. On the other hand, the hdcP gene is considered to be a histidine⁄ histamine antiporter [31]. The lack of hdcB and hdcRS indicates that they are not important to the histidine decarboxylation cascade and are likely to encode the accessory roles [34]. The integration of the histidine/histamine exchanger (hdcP) with a histidine decarboxylase (hdcA) forms a classical decarboxylation cascade in bacteria [8].

### 3.3. Microbiological Analyses of Smoked Horsemeat Sausages

The alterations in bacterial numbers are indicated in Table 3. LAB as well as GCC+ comprised the primary microbes in the fermentation as well as ripening given the elevated numbers of indigenous LAB and GCC+ in raw horsemeat coupled with their rapid growth. The *Enterobacteriaceae* initial numbers were more remarkably elevated in batches PMT and PO than in batch CK (*p* < 0.05), associated with the richness of *P. bacillus* in the initial population. In addition, the number of LAB and GCC+ in all batches rose sharply, reached the maximum on the seventh day, and then decreased slightly. Besides, there was no significant difference between the four smoked horsemeat sausage batches in the later period (*p* > 0.05). Thyme microcapsules have no significant bactericidal biological activity against LAB and GCC+ in all batches, which may be attributed to the fact that the sensitivity of microorganisms to thyme microcapsules varied from species to species [24]. This is consistent to findings documented by Lu et al. [5] on a similar pattern in LAB and GCC+ numbers in smoked horsemeat sausages augmented with plant extracts. The numbers of *Enterobacteriaceae* remarkably reduced in the fermentation as well as the ripening (*p* < 0.05). On day 28, *Enterobacteriaceae* was completely depleted in the PMT batch, while the count in the CK, P, and PO batches were 1.01 ± 0.03, 3.36 ± 0.01, 1.85 ± 0.029 log10 CFU g^−1^, respectively, revealing that thyme microcapsules repressed *Enterobacteriaceae* growth. Similarly, Scacchetti et al. [18] reported that thyme microcapsules showed high antimicrobial properties. The thyme microcapsule antibacterial mechanism is attributed to thyme EO slow release, which contains carvacrol, thymol, and other phenolic compounds, which are considered to be fungicides or antibacterial agents [35]. These components can attack the phospholipids in the cell membrane, increasing cell permeability, cytoplasmic leakage, or interaction with enzymes located on the cell wall to extend the shelf life of sausages in thyme microcapsule samples [17,18,35].

### 3.4. Gene Expression of hdcA and hdcP in Smoked Horsemeat Sausages

**Figure 2:**
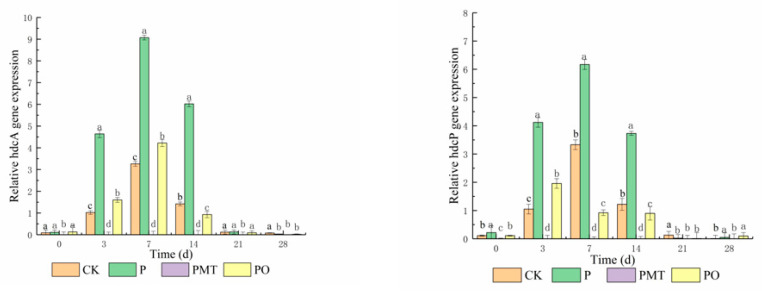
Effect of thyme microcapsules on gene expression of hdcA and hdcP in smoked horsemeat sausage during fermentation and ripening (average ± standard deviation, *n* = 3). (a–d): Values in the same column and batch not followed by a common letter are significantly different (*p* < 0.05) between days. Batch CK: the spontaneously fermented as the control; batch P: inoculated with *P. bacillus*; batch PMT: inoculated with *P. bacillus* and thyme microcapsules; batch PO: inoculated with *P. bacillus* and essential oil.

The hdcA and hdcP gene expression is involved in the production of histamine, which is found in our previous pure-culture system. The transcription of hdcA and hdcP genes in smoked horsemeat sausage is shown in Figure 2. The expression of hdcA and hdcP genes in all batches increased over the first seven days, then reached a maximum. There was a remarkable difference in the expression of hdcA and hdcP genes among the four batches (*p* < 0.05). The expression of the hdcA gene in batches CK, PO, and P was 9.58-, 18.64-, and 537.45-fold, respectively, which was higher compared to batch PMT. Similarly, the expression of the hdcp gene in batches CK, PO, and P was 10.06-, 1.89-, and 72.00-fold, respectively, which was higher than in batch PMT. Thyme microcapsules had a significantly higher effect on the expression of hdcA and hdcP compared to thyme oil. Studies have previously reported that the activity of the histamine synthesis gene cluster (HDC) affects the accumulation of histamine [31]. The factors affecting its activity include pH, temperature, salinity, and oxygen content, with pH being the main influencing factor. The acidic environment inhibits the growth of the test bacteria to a certain extent and affects the production and activity of enzymes. The expression of hdcA and hdcP genes increased under low pH conditions and the presence of extracellular histidine. Histamine accumulation by *P. bacillus* might confer resistance to the prevailing acidic stress via the consumption of intracellular protons through a decarboxylation reaction, as documented in other bacterial species [9,31,32]. Herein, the sausage processing conditions in all the samples were similar. Besides, the lack of significant difference in pH among the four batches showed that thyme microcapsules affected the activity of histidine decarboxylase and suppressed the expression of hdcA and hdcP genes.

### 3.5. Histamine Accumulation in Smoked Horsemeat Sausages

**Figure 3:**
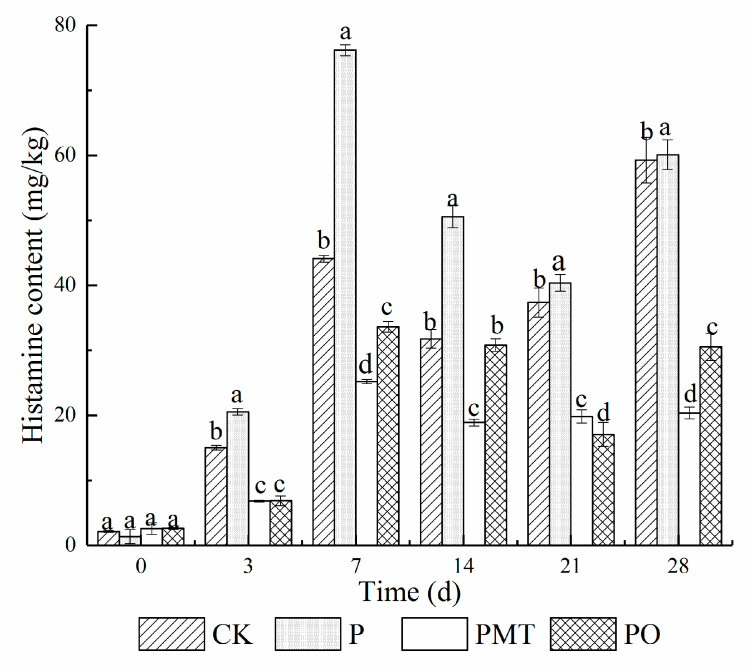
Effect of thyme microcapsules on histamine accumulation in smoked horsemeat sausage during fermentation and ripening (average ± standard deviation, *n* = 3). (a–d): Values in the same column and batch not followed by a common letter are significantly different (*p* < 0.05) between days. Batch CK: the spontaneously fermented as the control; batch P: inoculated with *P. bacillus*; batch PMT: inoculated with *P. bacillus* and thyme microcapsules; batch PO: inoculated with *P. bacillus* and essential oil.

The changes in histamine concentration in smoked horsemeat sausage are indicated in Figure 3. The histamine quantities in the four groups were measurable three days post fermentation and were found to increase during the first seven days, congruent with previous data in fermented sausages [24]. Higher levels of histamine (76.17, 35.18, and 44.09 mg/kg) were reported in the P, PO, and CK batches on day 7. On day 28, the histamine concentration in batches PMT and PO was 20.34 and 30.52 mg/kg, respectively, corresponding to a 66.15% and 49.22% decrease relative to batch P (*p* < 0.05) and suggesting that the presence of thyme oil prevented the accumulation of histamine. Compared with batch (PO), there was a 33.35% reduction in the accumulation of histamine in batch PMT, indicating that thyme microcapsule had a stronger inhibitory effect than thyme oil (*p* < 0.05). Additionally, thyme microcapsules decreased histamine aggregation by suppressing the expression of histidine decarboxylase (hdcA) as well as histidine/histamine antiporter (hdcP) genes (*p* < 0.05). Our data demonstrated that the encapsulation with thyme microcapsules reduced the accumulation of histamine, thus suggesting that the thyme microcapsule application can promote the safety of smoked horsemeat sausage as well as other meat products.

### 3.6. Sensory Quality

**Figure 4:**
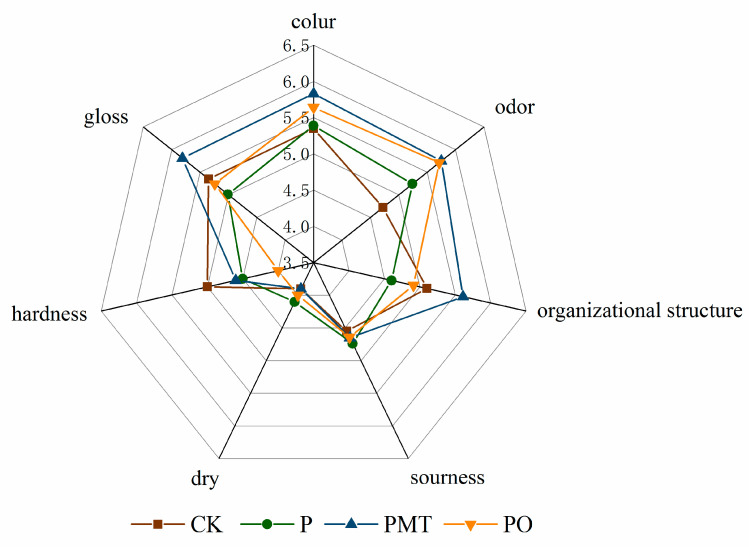
Sensory evaluation of smoked horsemeat sausage. Batch CK: the spontaneously fermented as the control; batch P: inoculated with *P. bacillus*; batch PMT: inoculated with *P. bacillus* and thyme microcapsules; batch PO: inoculated with *P. bacillus* and essential oil.

The sensory qualities of the smoked horsemeat sausages were assessed at day 28 and are shown in Figure 4. The sausages with thyme microcapsules had significantly higher score for color than other batches (CK, P, PO) (*p* < 0.05), which may be attributed to the presence of nitrite. Thyme microcapsules can lower the pH value, and under acidic conditions, more nitrosomyoglobin will be produced, which plays an important role in color development [36]. The gloss of fermented meat products is an important sensory attribute. There was lower score for gloss in the batch P, which may be attributed to the presence of a large number of microorganisms than other batches. Numerous factors impact the sensory quality of smoked horsemeat sausage, such as raw meat, microbial activity, pH value, and any other factors in sausage [37]. There was no remarkable difference (*p* > 0.05) in the sourness and dry scores among all sausage batches. Compared with the thyme EO group, the thyme microcapsules group had a better odor. Besides, thyme microcapsule as an encapsulated plant extract covered up the bad smell of thyme EO and also slowed down its release. This may also be attributed to the fact that thyme microcapsules reduce the accumulation of histamine. Ardö et al. [38] reported that though lipid oxidation is the primary cascade to the generation of flavor compounds, amino acid metabolism also plays a pivotal role in development of flavor. Additionally, compared to the batches P and PO, there were higher score for hardness and structure in the batch PMT. Some reports showed that the addition of EO microcapsule to fermented products can maintain tissue hardness and prolong the food shelf-life [35,39]. Therefore, adding thyme microcapsules can effectively inhibit the accumulation of histamine in smoked horsemeat sausage and can also improve its sensory quality.

## 4. Conclusions

Herein, we demonstrated that both thyme microcapsules and thyme essential oil (EO) could diminish histamine levels, down-regulate the expression of HDC gene cluster, and repress the microbial growth, e.g., *Enterobacteria* in smoked horsemeat sausage. Accumulation of histamine is not only linked to histamine-generating microbes but also associated with the histidine decarboxylase (HDC) gene cluster expression. Thyme microcapsules and thyme EO had insignificant influence on the expression of hdcRS and hdcB; however, they remarkably suppressed the transcriptional activation of hdcA as well as hdcP genes (*p* < 0.05). The suppressive influence of thyme microcapsules was remarkably higher (*p* < 0.05) relative to that in thyme EO. Additionally, the addition of thyme microcapsules enhanced the sensory quality of the smoked horsemeat sausage. Hence, the use of thyme microcapsules not only improves the smoked horsemeat sausage sensory quality but also enhances its safety.

## Figures and Tables

**Table 1:** Primers used in this work.

Gene	Primer	Sequence (5′→3′)
hdcA	hdcA-F	GATGGTATTGTTTCKTATGA
	hdcA-R	CCAAACACCAGCATCTTC
hdcP	hdcP-F	GTCTGATCCATGGACACGGCTGAAC
	hdcP-R	GTTGCCGCGAATCTAGAATC
hdcB	hdcB-F	TACCGTTAGAGGCGAGTTCC
	hdcB-R	GGCAGCACAGGATTAGCATC
hisRS	hisRS-F	CACACAGATTGGTTGTGAGGC
	hisRS-R	CGTCCCGTGTTTCTTTGTCAC
tuf	tuf-F	TCTTCATCATCAACAAGGTCTGCTT
	tuf-R	GAACACATCTTGCTTTCACGTCAA
recA	recA-F	CAAGGCTTAGAGATTGCCGATG
	recA-R	ACGAGGAACTAACGCAGCAAC

**Table 2:** Sensory standard of smoked horse sausage.

Attribute		Definitions (Developed by the Panel)
Appearance	color	The actual hue of the color, pink brown to dark brown
	gloss	Shiny, attractive marinated color and uniform color
	dry	The absence of moistness, resembling dried meat
Flavor	sourness	The smell of oranges
	odor	Scented with horse meat and marinated flavors
Texture	hardness	Tough and hard to chew
	organizational structure	Tissue is delicate and elastic

**Table 3:** Microbial counts (log 10 CFU g-1) during ripening of smoked horsemeat sausage.

Microbiological Counts	Batch	Days (d)				
		0	3	7	14	28
LAB	CK	3.86 ± 0.04 d	6.68 ± 0.01 c	7.19 ± 0.01 a	7.03 ± 0.02 b	7.11 ± 0.04 ab
	P	3.93 ± 0.07 d	6.81 ± 0.06 c	7.13 ± 0.01 ab	7.15 ± 0.02 a	7.09 ± 0.03 b
	PMT	3.91 ± 0.02 d	6.59 ± 0.02 c	7.09 ± 0.02 a	6.89 ± 0.01 b	7.01 ± 0.05 a
	PO	3.91 ± 0.03 e	6.66 ± 0.03 d	7.13 ± 0.02 b	6.92 ± 0.04 c	7.17 ± 0.08 a
GCC+	CK	3.97 ± 0.02 c	6.87 ± 0.01 b	7.79 ± 0.04 a	6.63 ± 0.08 b	6.54 ± 0.02 b
	P	3.95 ± 0.01 c	6.75 ± 0.04 b	7.46 ± 0.02 a	6.47 ± 0.03 b	6.45 ± 0.03 b
	PMT	3.95 ± 0.05 c	6.59 ± 0.06 b	7.79 ± 0.02 a	6.44 ± 0.04 b	6.26 ± 0.02 b
	PO	3.96 ± 0.03 c	6.61 ± 0.02 b	7.56 ± 0.02 a	6.43 ± 0.04 b	6.27 ± 0.07 b
Enterobacteria	CK	3.09 ± 0.07 a	3.16 ± 0.04 a	2.92 ± 0.02 b	2.11 ± 0.04 c	1.01 ± 0.01 d
	P	5.64 ± 0.05 a	4.92 ± 0.04 b	4.18 ± 0.06 c	3.97 ± 0.03 d	3.36 ± 0.01 e
	PMT	5.47 ± 0.02 a	4.31 ± 0.01 b	3.53 ± 0.02 c	3.09 ± 0.01 d	nd
	PO	5.59 ± 0.07 a	4.68 ± 0.02 b	3.96 ± 0.03 c	3.12 ± 0.01 d	1.85 ± 0.029 e

Batch CK: the spontaneously fermented as the control; batch P: inoculated with *P. bacillus*; batch PMT: inoculated with *P. bacillus* and thyme microcapsules; batch PO: inoculated with *P. bacillus* and essential oil; LAB, lactic acid bacteria; GCC+, gram-positive catalase-positive cocci. Data are expressed as mean ± standard deviation (*n* = 3). a–e: values in the same row and batch not followed by a common letter are significantly different (*p* < 0.05), differences between days. nd, not detected.

## Data Availability

The data presented in this study are available on request from the corresponding author. The data are not publicly available due to institutional privacy.

## References

[1] Ladero V., Calles-Enríquez M., Fernández M., Álvarez M.A. (2010). Toxicological effects of dietary biogenic amines. Curr. Nutr. Food Sci..

[2] Linares D.M., Del Río B., Ladero V., Martínez N., Fernández M., Martín M.C., Álvarez M.A. (2012). Factors influencing biogenic amines accumulation in dairy products. Front. Microbiol..

[3] Zhao L., Xue L., Li B., Wang Q., Li B., Lu S., Fan Q. (2018). Ferulic acid reduced histamine levels in the smoked horsemeat sausage. Int. J. Food Sci. Tech..

[4] Lin C., Tsai H., Lin C., Huang C., Kung H., Tsai Y. (2014). Histamine content and histamine-forming bacteria in mahi-mahi (*Coryphaena hippurus*) fillets and dried products. Food Control.

[5] Lu S., Ji H., Wang Q., Li B., Li K., Xu C., Jiang C. (2015). The effects of starter cultures and plant extracts on the biogenic amine accumulation in traditional Chinese smoked horsemeat sausages. Food Control.

[6] Lu S., Xu X., Shu R., Zhou G., Meng Y., Sun Y., Chen Y., Wang P. (2010). Characterization of biogenic amines and factors influencing their formation in traditional Chinese sausages. J. Food Sci..

[7] Bartkiene E., Bartkevics V., Mozuriene E., Krungleviciute V., Novoslavskij A., Santini A., Rozentale I., Juodeikiene G., Cizeikiene D. (2017). The impact of lactic acid bacteria with antimicrobial properties on biodegradation of polycyclic aromatic hydrocarbons and biogenic amines in cold smoked pork sausages. Food Control.

[8] Lucas P.M.W.W. (2005). Histamine-Producing Pathway Encoded on an Unstable Plasmid in Lactobacillus hilgardii 0006. Appl. Envirvron. Microb..

[9] Satomi M., Mori-Koyanagi M., Shozen K., Furushita M., Oikawa H., Yano Y. (2012). Analysis of plasmids encoding the histidine decarboxylase gene in Tetragenococcus muriaticus isolated from Japanese fermented seafoods. Fisheries Sci..

[10] Landete J.M., Pardo I., Ferrer S. (2008). Regulation of hdc expression and HDC activity by enological factors in lactic acid bacteria. J. Appl. Microbiol..

[11] Satomi M., Furushita M., Oikawa H., Yoshikawatakahashi M., Yano Y. (2008). Analysis of a 30 kbp plasmid encoding histidine decarboxylase gene in Tetragenococcus halophilus isolated from fish sauce. Int. J. Food Microbiol..

[12] Pereira C.I., Crespo M.T., Romao M.V. (2001). Evidence for proteolytic activity and biogenic amines production in Lactobacillus curvatus and *L. Homohiochii*. Int. J. Food Microbiol..

[13] Tapingkae W., Tanasupawat S., Parkin K.L., Benjakul S., Visessanguan W. (2010). Degradation of histamine by extremely halophilic archaea isolated from high salt-fermented fishery products. Enzyme Microb. Tech..

[14] Wang Y., Li F., Zhuang H., Chen X., Li L., Qiao W., Zhang J. (2015). Effects of plant polyphenols and α-tocopherol on lipid oxidation, residual nitrites, biogenic amines, and N-nitrosamines formation during ripening and storage of dry-cured bacon. LWT.

[15] Burt S. (2004). Essential oils: Their antibacterial properties and potential applications in foods—A review. Int. J. Food Microbiol..

[16] Van Haute S., Raes K., Van der Meeren P., Sampers I. (2016). The effect of cinnamon, oregano and thyme essential oils in marinade on the microbial shelf life of fish and meat products. Food Control.

[17] Hu J., Zhang Y., Xiao Z., Wang X. (2018). Preparation and properties of cinnamon-thyme-ginger composite essential oil nanocapsules. Ind. Crop. Prod..

[18] Scacchetti F.A.P., Pinto E., Soares G.M.B. (2017). Functionalization and characterization of cotton with phase change materials and thyme oil encapsulated in beta-cyclodextrins. Prog. Org. Coat..

[19] Simon-Brown K., Solval K.M., Chotiko A., Alfaro L., Reyes V., Liu C., Dzandu B., Kyereh E., Goldson Barnaby A., Thompson I. (2016). Microencapsulation of ginger (*Zingiber officinale*) extract by spray drying technology. LWT.

[20] Fernández E., Alegría Á., Delgado S., Martín M.C., Mayo B. (2011). Comparative Phenotypic and Molecular Genetic Profiling of Wild *Lactococcus lactis* subsp.lactis Strains of the *L. lactis* subsp. lactis and *L. Lactis* subsp. cremoris Genotypes, Isolated from Starter-Free Cheeses Made of Raw Milk. Appl. Environ. Microb..

[21] Linares D.M., Alvarez-Sieiro P., Del Rio B., Ladero V., Redruello B., Martin M.C., Fernandez M., Alvarez M.A. (2015). Implementation of the agmatine-controlled expression system for inducible gene expression in *Lactococcus lactis*. Microb. Cell Fact..

[22] Del Rio B., Linares D.M., Ladero V., Redruello B., Fernández M., Martin M.C., Alvarez M.A. (2015). Putrescine production via the agmatine deiminase pathway increases the growth of *Lactococcus lactis* and causes the alkalinization of the culture medium. Appl. Microbiol. Biot..

[23] Lu S., Xu X., Zhou G., Zhu Z., Meng Y., Sun Y. (2010). Effect of starter cultures on microbial ecosystem and biogenic amines in fermented sausage. Food Control.

[24] Laranjo M., Gomes A., Agulheiro-Santos A.C., Potes M.E., Cabrita M.J., Garcia R., Rocha J.M., Roseiro L.C., Fernandes M.J., Fraqueza M.J. (2017). Impact of salt reduction on biogenic amines, fatty acids, microbiota, texture and sensory profile in traditional blood dry-cured sausages. Food Chem..

[25] Del Rio B., Redruello B., Ladero V., Fernandez M., Martin M.C., Alvarez M.A. (2016). Putrescine production by *Lactococcus lactis* subsp. Cremoris CECT 8666 is reduced by NaCl via a decrease in bacterial growth and the repression of the genes involved in putrescine production. Int. J. Food Microbiol..

[26] Livak K.J., Schmittgen T.D. (2001). Analysis of relative gene expression data using real-time quantitative PCR and the 2(-Delta Delta C(T)) Method. Methods.

[27] Sun Q., Zhao X., Chen H., Zhang C., Kong B. (2018). Impact of spice extracts on the formation of biogenic amines and the physicochemical, microbiological and sensory quality of dry sausage. Food Control.

[28] Park J.S., Lee C.H., Kwon E.Y., Lee H.J., Kim J.Y., Kim S.H. (2010). Monitoring the contents of biogenic amines in fish and fish products consumed in Korea. Food Control.

[29] Dansby M.A., Bovell-Benjamin A.C. (2003). Sensory characterization of a ready-to-eat sweetpotato breakfast cereal by descriptive analysis. J. Food Sci..

[30] Bel Hadj Salah-Fatnassi K., Hassayoun F., Cheraif I., Khan S., Jannet H.B., Hammami M., Aouni M., Harzallah-Skhiri F. (2017). Chemical composition, antibacterial and antifungal activities of flowerhead and root essential oils of *Santolina chamaecyparissus* L., Growing wild in Tunisia. Saudi J. Biol. Sci..

[31] Hsu H., Chuang T., Lin H., Huang Y., Lin C., Kung H., Tsai Y. (2009). Histamine content and histamine-forming bacteria in dried milkfish (Chanos chanos) products. Food Chem..

[32] Kimura B., Takahashi H., Hokimoto S., Tanaka Y., Fujii T. (2009). Induction of the histidine decarboxylase genes of Photobacterium damselae subsp. Damselae (formally *P. histaminum*) at low pH. J. Appl. Microbiol..

[33] Satomi M., Furushita M., Oikawa H., Yano Y. (2011). Diversity of plasmids encoding histidine decarboxylase gene in *Tetragenococcus* spp. Isolated from Japanese fish sauce. Int. J. Food Microbiol..

[34] Calles-Enriquez M., Eriksen B.H., Andersen P.S., Rattray F.P., Johansen A.H., Fernandez M., Ladero V., Alvarez M.A. (2010). Sequencing and transcriptional analysis of the streptococcus thermophilus histamine biosynthesis gene cluster: Factors that affect differential hdcA expression. Appl. Environ. Microb..

[35] Xiao Z., Kang Y., Hou W., Niu Y., Kou X. (2019). Microcapsules based on octenyl succinic anhydride (OSA)-modified starch and maltodextrins changing the composition and release property of rose essential oil. Int. J. Biol. Macromol..

[36] Flores M., Toldrá F. (2011). Microbial enzymatic activities for improved fermented meats. Trends Food Sci. Tech..

[37] Coloretti F., Chiavari C., Poeta A., Succi M., Tremonte P., Grazia L. (2019). Hidden sugars in the mixture: Effects on microbiota and the sensory characteristics of horse meat sausage. LWT.

[38] Ardö Y. (2006). Flavour formation by amino acid catabolism. Biotechnol. Adv..

[39] Saelao S., Maneerat S., Thongruck K., Watthanasakphuban N., Wiriyagulopas S., Chobert J., Haertlé T. (2018). Reduction of tyramine accumulation in Thai fermented shrimp (kung-som) by nisin Z-producing *Lactococcus lactis* KTH0-1S as starter culture. Food Control.

